# Role of Nonpharmaceutical Interventions during 1918–1920 Influenza Pandemic, Alaska, USA

**DOI:** 10.3201/eid3107.241048

**Published:** 2025-07

**Authors:** Uddhav Khakurel, Lisa Sattenspiel, Svenn-Erik Mamelund

**Affiliations:** Centre for Research on Pandemics & Society, Oslo Metropolitan University, Oslo, Norway (U. Khakurel, S.-E. Mamelund); University of Missouri, Columbia, Missouri, USA (L. Sattenspiel)

**Keywords:** Influenza, respiratory infections, viruses, pandemic, nonpharmaceutical interventions, isolated areas, remoteness, indigenous peoples, Alaska, United States

## Abstract

Previous studies investigating the 1918–1920 influenza pandemic have provided a comprehensive overview of the spread of the pandemic and possible explanations for high mortality rates in Alaska, USA. Our understanding of the role of nonpharmaceutical interventions (NPIs) is limited, however. To gain an overview of various agencies’ efforts to protect communities during the pandemic, we conducted a mixed-method assessment of a large pool of digitized historical newspapers and archival materials covering Alaska’s local and territorial responses to the pandemic. The study encompassed 14 local units of Alaska that implemented NPIs during October 1918–January 1919. Analyses indicated that 8 local units avoided the outbreak by implementing NPIs and that the other 6 units controlled the spread of influenza by implementing NPIs after the virus was introduced. In addition, some Indigenous communities escaped the pandemic by implementing mandatory and voluntary restrictions. Information on the effects NPI of could guide future influenza pandemic preparedness and response.

During the 1918–1920 influenza pandemic, although scientists were aware of the existence of infectious agents smaller than bacteria (*1*), they thought that the *Hemophilus influenzae* bacterium was responsible for causing the disease. Influenza was not identified as a virus in swine until 1931 and in humans in 1933 (*2*,*3*). In 1918, however, public health officials and healthcare workers were aware that influenza spread through the air (*4*). The national and local authorities of Canada, Australia, and the United States attempted to implement different nonpharmaceutical interventions (NPIs), including travel restrictions and quarantine, to prevent the spread of the influenza pandemic (*5*). The primary focus in those countries was to avoid the worst impacts of the pandemic by directing efforts toward implementing protective measures, such as establishing quarantine stations and stopping travel between influenza-infected and uninfected communities (*6*).

However, quarantines did not prevent introduction of influenza in all communities (*7*). Once the disease was introduced, large cities across the United States implemented a wide range of NPIs to limit community-level influenza transmission. Community-level interventions included isolation, school closures, public gathering bans, and surface cleaning (*8*–*10*). Local efforts to contain the spread of the infectious agent were derived from cities’ experience in managing outbreaks of tuberculosis, cholera, and smallpox in the 18th and 19th Centuries (*1*).

During the 1918–1920 influenza pandemic, Alaska was a US territory. The territorial government of Alaska was forewarned about the risk for an influenza outbreak and implemented quarantine regulations to prevent the introduction of the disease (*11*). The territorial governor ordered all communities to establish quarantines and to create cordon sanitaire (protective buffer zones), by limiting travel at trailheads and along rivers (*12*–*15*). A previous report showed that communities and residents adopted different community-level NPIs to control the spread of influenza (*8*). Those interventions included travel restrictions, quarantine of travelers, isolation, prohibition of public gatherings and native festivals, fumigation of public places, and school closures. However, implementing NPIs did not prevent introduction of influenza into Alaska (*8*). The 1918–1920 influenza pandemic killed at least 50 million persons worldwide, including 675,000 persons in the United States (*12*). In Alaska, the average influenza mortality rate ranged from 1% to 38% at the regional level, and some local communities reported mortality rates of >90% (*8*,*16*).

Researchers have previously investigated the role of NPIs in 1918–1920 influenza mortality rates in urban settings (*9*). Those studies focused on the larger US cities and concluded that, although implementation of NPIs did not stop the spread of the pandemic, it helped delay its spread, known contemporarily and colloquially as flattening the curve (*17*). Peak mortality rates in cities under NPI use was lower than in cities with no reported NPIs (*9*).

Little previous research has considered the effects of NPIs on local-level variation in influenza spread and mortality rates in geographically isolated areas like Alaska. One study discussed several factors that influenced patterns of influenza deaths in Alaska and Labrador (*11*), an isolated region in Canada with a similar culture and latitude to Alaska. That study highlighted the cocirculation of other pathogens, environmental influences, and access to healthcare but did not consider the effects of NPIs implemented by the local board of health and territorial government on the variation in mortality rates across the 2 regions.

We investigated the role of NPIs in reducing the spread of the influenza during the 1918–1920 pandemic and on pneumonia and influenza (P&I) mortality rates in Alaska. We focused on the first wave of influenza in Alaska, during September 1918–January 1919 because evidence for the implementation of NPIs in later waves is lacking. We investigated the type and duration of interventions implemented at local levels, patterns of influenza spread, daily mortality rates in Alaska, and types and effects of NPIs implemented by Alaska Native populations.

## Methods

### Study Context

In 1910, Alaska was inhabited by 64,356 persons in an area of 1.72 million km^2^ and had a predominantly male (71.25%) population. During the 1918–1920 influenza pandemic, the territory was divided into 4 judicial districts (First, Second, Third, and Fourth) and 42 local units. The territorial headquarters was located in Juneau in the First Judicial District. From 1910 to 1920, the population of Alaska dropped by 14.5% to 55,036 (*18*). The Alaska Native population numbers were similar, at ≈27,000, in both 1910 and 1920 (*16*).

Influenza in Alaska came in 3 distinct waves (*8*,*16*). The first wave started in October 1918 and corresponded with the second worldwide influenza wave. In some communities, the first wave continued until January 1919 (*12*). Influenza was introduced into Alaska by persons entering and leaving trading and fishing vessels or coastal steamers (*8*). A similar observation was made in the second influenza wave in Alaska (the third worldwide wave), which started in May 1919. A small third wave occurred in Alaska in 1920 (*16*). 

**Figure 1:**
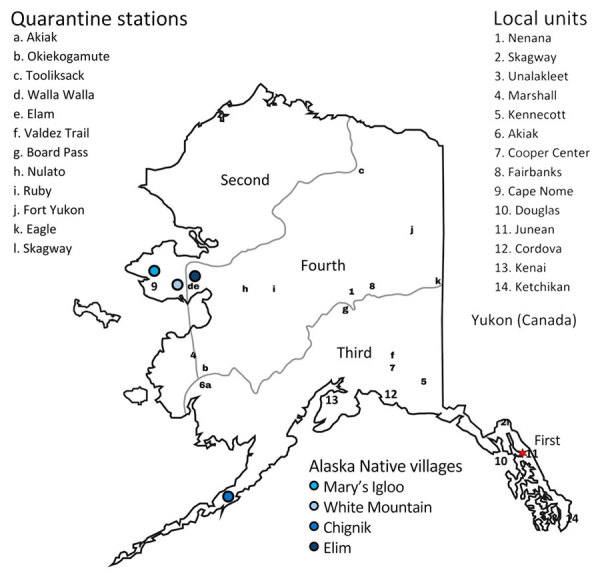
Locations of Judicial Districts, quarantine stations, native villages, and local units included in study of the role of nonpharmaceutical interventions during 1918–1920 influenza pandemic, Alaska, USA. Quarantine stations were located along the Iditarod, Valdez, Yukon, Innoko River, and Fairbanks Trails at Elam, Akiak, Okiekogamute, Tooliksack, Walla Walla, Piledriver, Board Pass, Nulato, Ruby, Fort Yukon, Eagle, and Skagway. This study includes data from Cape Nome, Douglas, Juneau (territorial headquarters; denoted by red star), Cordova, Kenai, Ketchikan, Nenana, Skagway, Unalakleet, Marshall, Kennecott, Akiak, Copper Center, and Fairbanks.

We examined the role of NPIs in 14 local units and 4 Alaska Native villages during the first influenza wave, October 1918–January 1919, for which we have information on the pandemic and NPIs (Figure 1). We focused on the first wave of influenza because evidence for the implementation of NPIs in later waves is lacking. The limited information available for subsequent waves might be attributed to pandemic fatigue, a phenomenon observed during the COVID-19 pandemic (*19*). However, definitive evidence on pandemic fatigue in Alaska during the 1918–1920 influenza pandemic has not been identified.

### Data Sources

We collected historical data on the influenza pandemic and NPIs implemented in Alaska during 1918–1920 from the digital archive of the Library of Congress (https://www.loc.gov) and the National Archives Catalog (https://catalog.archives.gov). Photographs and newspaper articles, a primary source of information in this study, are available in the digital archive of the Library of Congress. Government letters, announcements, reports, and regulations are available in the National Archives Catalog. We collected additional materials from the Alaska State Archives (https://archives.alaska.gov). We used all local newspapers from the first influenza wave in Alaska that were available in the digital archive (Table 1). We screened a total of 15 newspapers to identify the types of interventions implemented at the local level. Those records contained information on the influenza pandemic in Alaska and NPIs implemented by the territorial and local governments and health boards. Further, we extracted information on quarantine expenses and reimbursement, hiring of quarantine officers, arrival of steamships, and NPIs adopted by Indigenous communities from the Alaska newspaper articles and additional archival resources.

**Table 1:** List of newspapers used to investigate the role of nonpharmaceutical interventions during 1918–20 influenza pandemic, Alaska, USA

Local newspapers	City or town
Nome Tri-Weekly Nugget	Nome
Daily Progressive Miner	Ketchikan
Cordova Daily Times	Cordova
Alaska Daily Empire	Juneau
The News Letter	Kodiak
Alaska Juneau Douglas Island News	Juneau
Seward Gateway Daily Edition	Seward
The Alaska Weekly Post	Seward
Seward Gateway	Seward
Douglas Island News	Douglas City
Nenana Daily News	Nenana
Weekly Alaska Citizen	Fairbanks
Wrangell Sentinel	Wrangell
McCarthy Weekly News	McCarthy
Weekly Nome Industrial	Nome

We obtained death certificates from Health Analytics and Vital Records, Division of Public Health, State of Alaska Department of Health. A total of 2,390 death certificates were recorded during October 1918–January 1919, of which 1,024 were P&I deaths and were included in the study. Death certificates include information on the place of death, sex, race, birth date, and cause of death and cover all parts of the Alaska territory. We excluded 51 death certificates from the study because the cause of death was missing. The full analysis of the death certificates was done in a previous study (*16*).

### Data Analysis

We used a mixed-method approach to analyze the role of NPIs in the spread of the influenza pandemic and the effects of NPIs on P&I mortality rates. We categorized NPIs implemented by authorities into preventive and spread control (Table 2). Preventive interventions were implemented to prevent the introduction of influenza in the community, also known as protective sequestration (*6*). We defined spread control NPIs as the interventions implemented after the introduction of influenza in the community (*9*). We further categorized spread control NPIs into 4 groups on the basis of the categorization used for the 1918 influenza pandemic (Table 2), which reflect the understanding of the authorities from 1918 (*9*,*20*,*21*). By comparison, the COVID-19 pandemic categorized NPIs into >13 categories using more granular information (*22*–*24*).

**Table 2:** Nonpharmaceutical interventions used during 1918–20 influenza pandemic, Alaska, USA

Preventive NPIs	Spread control interventions
Travel restriction to the community	Quarantine and isolation: orders to separate ill persons and persons suspected of having contact with ill persons
	School closures: closure of all schools under the jurisdiction of local authorities
Quarantine, >5 days	Public gathering bans: closure of saloons, restaurants, indoor gatherings, sports halls, public libraries, and local festivals
	Personal and ancillary actions: mask ordinance, fumigation of mail, and cleaning of surfaces

We measured NPI duration from the day the intervention began to the day it was lifted (Table 3). To study the role of NPIs in P&I deaths in Alaska, we examined the P&I deaths in the local units. We compared the mortality rate among local units that implemented NPIs with Alaska’s overall average regional mortality rate. We investigated the interventions the local units applied and the spread of the pandemic. We calculated the reproduction number (R) for all of Alaska and the local units that implemented NPIs after the first reported P&I death. Finally, we examined the use of NPIs among Alaska Natives. 

**Table 3:** Local units with spread control NPIs in a study of the role of nonpharmaceutical interventions during 1918–1920 influenza pandemic, Alaska, United States

Location	1910 population	No. deaths	Period of NPI (start–end)	Types of NPIs
Cape Nome	3,924			
Phase 1		0	4 d (1918 Nov 20–23)	Quarantine
Phase 2		334	63 d (1918 Nov 4–1919 Jan 6)	School closure, public gathering ban, quarantine, and isolation
Douglas	1,722			
Phase 1		0	41 d (1019 Oct 30–Dec 9)	School closure, public gathering ban, quarantine, and isolation
Phase 2		6	16 d (1918 Dec 19–1919 Jan 3)	School closure, public gathering ban, quarantine, isolation, and mandatory mask ordinance
Juneau	2,910			
Phase 1		19	33 d (1918 Oct 29–Dec 1)	School closure, public gathering ban, quarantine, and isolation
Phase 2		8	13 d (1918 Dec 18–30)	School closure, public gathering ban, quarantine, isolation, and mandatory mask ordinance
Cordova	1,779			
Phase 1		6	25 d (1918 Dec 27–1919 Jan 20)	School closure, public gathering ban, quarantine, and isolation
Kenai	1,692			
Phase 1		24	28 d (1918 Nov 8–Dec 5)	School closure, public gathering ban, quarantine, and isolation
Ketchikan	3,520			
Phase 1		21	30 d (1918 Oct 23–Nov 22)	School closure, public gathering ban, quarantine, and isolation

### Estimating R

We estimated R from the early growth phase of the epidemic trajectories by using the GrowthPredict toolbox (*25*) and mortality data from Alaska. GrowthPredict is a user-friendly tool designed for analysis of outbreak trajectories that accommodates subexponential growth patterns often observed in real-world situations. To calculate R, we extracted the mortality data for the first 20 days after the first reported P&I death in the local units. For that analysis, we excluded Cape Nome because the community did not introduce NPIs until after P&I deaths were reported among its population. For the calculation, we used 3 as an estimation of the serial interval for influenza on the basis of previous literature (*26*).

GrowthPredict toolbox uses the generalized growth model and characterizes the growth rate (r) and growth parameter (p). The growth parameter modulates the growth from purely exponential (p = 1) to subexponential (0<p>1). In our study, we used maximum-likelihood estimation to determine the best fit of the observed data.

We calculated R with the relationship defined in the renewal equation (*26*). The renewal equation incorporated the generation time distribution of influenza, which represents the time interval between successive infections in a transmission chain (*26*). We determined the generation time by using data from existing epidemiologic literature (*26*), which we input into the toolbox as a fixed parameter. The GrowthPredict toolbox provides CIs for R by performing parametric bootstrapping, generating multiple resampled datasets, and re-estimating R for each resample to account for statistical uncertainty (*25*).

## Results

Data available from archival resources indicated that the territorial government acted promptly to attempt to prevent the onset of influenza in the whole of Alaska. Once influenza was introduced, local governments and health boards implemented NPIs to protect persons living within their jurisdiction.

### Introduction of NPIs in Alaska

The territorial government of Alaska was warned about influenza spreading across the United States in late September 1918. In mid-October 1918, the US Public Health Department sent an official health bulletin to Governor Riggs with details on the incubation period, mode of transmission, and preventive measures (*27*). In response to the bulletin, in early October, the territorial government introduced quarantine regulations at port towns, including Juneau, Cordova, Seward, Valdez, and Cape Nome, to prevent the introduction of influenza into Alaska from incoming steamships:

Quarantine is established by the Governor against all incoming steamers and other marine craft on October 15th. All vessels are met at arrival at any port by designated physicians and should cases of influenza be found immediate action is taken [The Alaska Daily Empire (Juneau), October 24, 1918] (*28*).

A special request was made to steamship companies to examine all passengers for influenza. Those examinations were focused on close inspection of the nose and throat, with an emphasis on evidence of inflammation, and the gathering of information about exposure to infection.

### Spread Control NPIs

**Figure 2:**
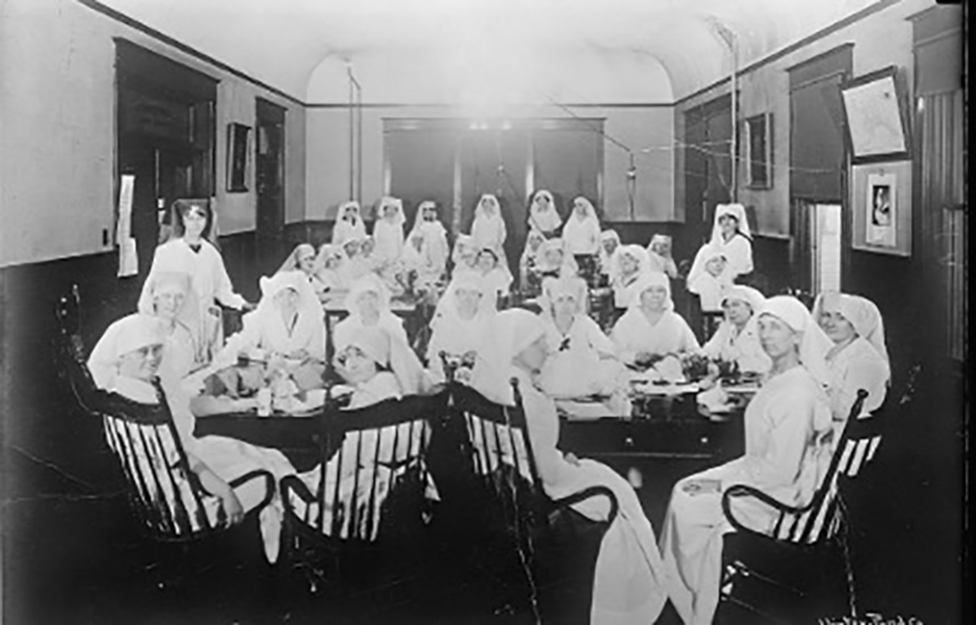
Archival photo of Juneau Chapter of the Red Cross from study of nonpharmaceutical interventions during 1918–1920 influenza pandemic, Alaska, USA. During the pandemic, the Red Cross made cloth mask and distributed to populations in Alaska. However, their reach was limited. Source: Library of Congress (https://www.loc.gov/resource/anrc.06015).

On the first appearance of influenza at the local level, the territorial government requested that local units implement interventions to prevent the spread of the disease. Cape Nome, Douglas, Juneau, Cordova, Kenai, and Ketchikan implemented school closures, public gathering bans, and quarantine and travel restrictions (Table 3). Those local units further tried to restrict the transmission of influenza to neighboring communities by restricting outgoing travel, commonly known as cordon sanitaire (*29*). Mask use was recommended through an official health notice. The Red Cross made and distributed cloth masks to populations in Alaska (Figure 2), but their reach was limited. 

Cape Nome introduced a short quarantine when the last steamship of the season, *Victoria*, arrived on October 20, 1918. The containment measure was lifted on October 23, 1918, because the local health officer did not record any symptoms of influenza among the incoming passengers (*30*). The early lifting of quarantine resulted in several P&I deaths in Cape Nome. After November 3, 1918, several deaths with P&I listed as a primary cause were recorded. The local board of health again implemented NPIs on November 4, 1918, a measure that lasted for 63 days and was lifted on January 6, 1919.

Douglas, a community located in the First Judicial District, isolated itself from late October to early December with quarantine regulations, school closures, and public gathering bans. After lifting the restrictions, Douglas reported P&I deaths on December 9, 1918, after which NPIs were again introduced along with mandatory mask ordinances (ancillary measures), which they strictly enforced:

Several arrests were made in Douglas yesterday on account of not wearing face masks. After one or two citizens had been fined, everyone began to see that officials meant business and after the second trip of the ferry everyone on the streets were wearing the ‘bug catchers’ [The Alaska Daily Empire (Juneau), November 13, 1918] (*31*).

The local units of Alaska maintained NPIs until local health officers reported no new influenza cases within the community and neighboring communities, at which point they lifted restrictions (*32*). A total of 1,024 P&I deaths during the first influenza wave were reported from the parts of the First, Second, and Third Judicial Districts, an aggregate population of 27,667 persons. Besides Cape Nome, which reported high (8.5%) mortality rates, the other local units with known NPIs reported low (1%–2%) P&I mortality rates compared with the territorial average of 3.7%, including local units with reported P&I deaths. Similarly, R for local units implementing NPIs, apart from Cape Nome, was lower (R = 0.31 [95% CI 0.26–0.89]) than the overall R for the whole territory (R = 0.92 [95% CI 0.87–0.97]).

### Preventive NPIs

Eight local units escaped the pandemic during Alaska’s first influenza wave (Table 4). Although those units were well connected with the communities experiencing influenza outbreaks via sled roads, pack trails, and railroads (*33*), they did not report P&I deaths during September 1918–February 1919. Those local units implemented protective sequestration measures by imposing travel restrictions, establishing quarantine stations along the trails leading to their communities (Figure 1), placing armed guards at the quarantine stations, and not allowing anyone to enter their communities beginning in early November 1918 (*34*).

**Table 4:** Communities with preventive NPIs in a study of the role of nonpharmaceutical interventions during 1918–1920 influenza pandemic, Alaska, United States*

Place	1910 population†	No. days NPI implemented (start–end dates)	Types of NPIs
Nenana	190	53 (1918 Nov 8–Dec 31)	Travel restriction and quarantine
Skagway	872	110 (1919 Nov 2–1919 Feb 20)	Travel restriction
Unalakleet	247	96 (1918 Nov 6–1919 Feb 10)	Quarantine
Marshall	NA	14 (1919 Jan 24–Feb 7)	Travel restriction
Kennecott	NA	42 (1918 Dec 21–1919 Feb 1)	Travel restriction and quarantine
Akiak	NA	35 (1919 Jan 8–Feb 12)	Travel restriction
Copper Center	553	75 (1918 Dec 18–March 1919)‡	Quarantine
Fairbanks	3,511	80 (1918 Nov 11–1919 Jan 27)	Travel restriction and quarantine

*NA, not available; NPIs, nonpharmaceutical interventions. †Marshall, Kennecott, and Akiak were not reported in the 1910 population census. ‡Minimum estimate.

Nenana adopted a unique approach by requiring residents to wear a red ribbon in their headgear to indicate that they were free from influenza:

The quarantine regulations at Nenana require the dwellers of that town to wear a red, green, blue yellow or other badge displayed upon their headgear. … The red badge is the indication that the wearer is free from such disease… dire penalties – fine and imprisonment – are threatened all who do not wear them prominently displayed on their headpieces [The Cordova Daily Times. December 23, 1918] (*35*).

Fairbanks used a similar strategy, in which health authorities examined residents periodically for influenza and gave an “OK Fairbanks Health Department” band to wear to indicate that the person was free from influenza.

### NPIs among Alaska Natives

**Figure 3:**
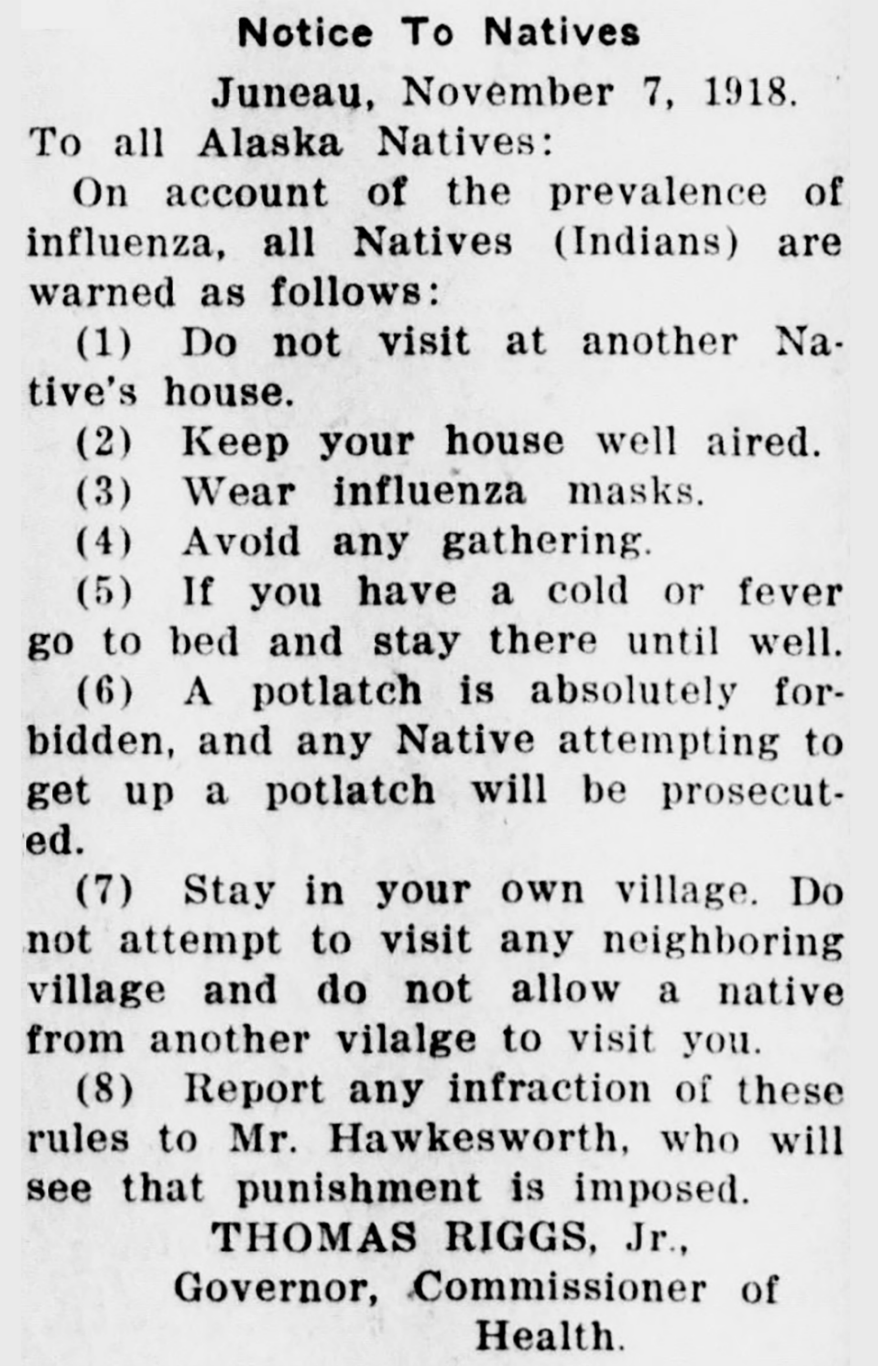
News clipping used in study of the role of nonpharmaceutical interventions during 1918–1920 influenza pandemic, Alaska, USA. This clipping from The Alaska Daily Empire, Juneau, dated November 7, 1918, is an example of nonpharmaceutical interventions imposed by the territorial government against Alaska Natives. A potlatch is an Alaska Native festival mostly celebrated in southeastern Alaska. Source: Library of Congress.

Alaska Natives experienced high mortality rates during the first influenza wave in the territory. Local newspapers repeatedly reported that Alaska Natives were at higher risk for the disease (*36*,*37*). The territorial government implemented stringent measures against public gatherings among Alaska Natives. The directive forwarded by the territorial government urged Alaska Natives not to visit neighbors, to keep houses well aired, to wear influenza masks, and to avoid gatherings; it also banned potlatch, a native festival mostly celebrated in southeastern Alaska (Figure 3).

In addition to those regulations, Alaska Natives implemented voluntary quarantine measures to safeguard themselves from influenza. The introduction of voluntary quarantine protected the Alaska Native villages of White Mountain, Elim, and Chignik (Figure 1). At Mary’s Igloo, Alaska Natives coming from Teller spread influenza among residents of the lower part of the settlement:

Chena Indians are enforcing quarantine regulations against natives from farther down the Tanana River [that river crosses through Judicial Districts 3 and 4 and passes close to Fairbanks and Nenana] [The Nome Tri-Weekly Nugget. April 18, 1919] (*38*).

Local teachers immediately enforced a quarantine measure to prevent the spread of influenza to the upper part of the settlement.

## Discussion

Despite being geographically isolated, the government of Alaska became aware of the imminent threat of an influenza outbreak in early October 1918, which enabled authorities to implement protective measures. Contrary to reports from other geographically isolated areas like American Samoa, New Caledonia, and Rotuma, where the forewarned governments were able to protect their inhabitants during the 1918–1920 influenza pandemic, influenza began spreading quickly in Alaska in late October (*10*,*39*–*41*). The first P&I death occurred in Cape Nome after the early lifting of quarantine because the health officer observed no symptoms among passengers quarantined from a steamship. Similar observations were made in Fiji and Tahiti in French Polynesia in 1918, where authorities released asymptomatic steamship passengers without quarantine and saw an influenza outbreak in the community (*10*).

Researchers have mentioned that infected persons can transmit diseases like influenza 24–48 hours before showing symptoms (*42*). A similar spread might have contributed to the introduction and subsequent large influenza outbreak in Cape Nome. NPIs were reintroduced only after a reported influenza death in the community. Similar observations were made in Douglas where the local authorities prevented an outbreak until early December 1918. The lifting of the quarantine resulted in a few cases in the middle of December, but Douglas reported only a few P&I deaths. In addition, except for Cape Nome, local units with NPIs had low R compared with the whole of Alaska. The increased awareness among residents during that period might have contributed to their adoption of precautionary measures against the subsequent outbreak. Despite repeated reporting by local newspapers on precautionary measures, available information is not sufficient to draw definitive conclusions.

Douglas, Juneau, Cordova, Kenai, and Ketchikan implemented influenza-informed NPIs during the pandemic (Table 3). Communities implemented NPIs on the basis of the risk for new cases as assessed by the health officer and local authorities. Researchers have concluded that low mortality rates were achieved in large cities that had long and sustained NPI implementation (*9*,*21*). In Alaska, we found that local units that implemented NPIs had lower mortality rates and reproduction numbers compared with the average mortality rate at the territorial level, which included all areas with reported influenza deaths. Although this study does not allow for a direct comparison of mortality rates between local units with varying levels of NPI use, the findings offer valuable insights into the role of NPIs in P&I mortality rates. Furthermore, the lower mortality rate in the First Judicial District might be related to greater access to healthcare (*8*,*11*). In addition, the role of NPIs in flattening the morbidity curve has been well understood from the 1918 influenza pandemic and the COVID-19 pandemic (*9*,*17*,*43*,*44*) and might have contributed to the low mortality rate in the First Judicial District.

Eight Alaska communities implemented quarantine regulations and travel restrictions that protected them from the influenza outbreak and helped stop spread of the virus into the interior of Alaska. Similar observations were made in 6 US communities where the local authorities implemented protective measures and took advantage of geographic isolation (*6*). As explained in earlier studies on the pandemic in Alaska, the mobility of the population during the fall of 1918 was limited because of rugged geography and lack of snow during the fall of that year (*8*,*11*). That limited mobility likely contributed to the effectiveness of quarantine regulations (*45*). In addition, Fairbanks and Nenana introduced an identification mechanism to separate infected and uninfected persons, comparable to COVID-19 vaccine certificates issued in many countries during that pandemic (*46*).

Indigenous communities worldwide faced high mortality rates during the 1918–1920 influenza pandemic (*8*,*47*–*49*). The local governments at the time were aware of the elevated influenza risk and implemented stringent measures to protect Indigenous communities. Our findings argue that implementing strict measures prevented influenza from reaching all Indigenous communities, which complements the results from previous research in South Pacific islands (*10*). Indigenous communities living in Alaska also implemented protective measures through voluntary initiatives. Although this finding is specific to the 1918–1920 influenza pandemic, researchers noted similar observations from around the globe during the COVID-19 pandemic (*50*).

The first limitation of this study is that we could only include 14 local units in Alaska because of a lack of data. Second, the level of travel to Alaska’s interior during the winter months is yet to be explored in full detail. Because transmission relies on human contact, we could only include the parts of Alaska with complete information on NPIs and travel. Finally, we were not able to include public opinion and opposition or adherence to the NPIs and the possible effects of those actions on P&I mortality rates. Future explorations of the 1918–1920 influenza pandemic in Alaska will be directed toward those issues.

Although several previous studies have focused on the city-to-city variation in mortality rates in the continental United States, this study provides insight into the role of NPIs in geographically isolated areas in Alaska and the role of NPIs in limiting the spread of influenza. Results from the study suggest that the territorial government made efforts to prevent the spread of the pandemic in Alaska. The eventual spread of the pandemic in October 1918, however, led the local governments to implement NPIs. School closures, public gathering bans, and quarantine and isolation were the main NPIs used in Alaska. The lower R for the areas with NPIs further provides quantitative evidence that NPIs helped to protect the communities. This study supports previous studies concluding that protective sequestration measures protect isolated communities. Further, through this study, we found additional evidence of Alaska Natives adopting voluntary NPIs.

To capture the overall picture of entire territory of Alaska, further work will examine other written sources from the time, such as diaries and letters, to investigate whether more information can be sourced. The oral traditions of the Indigenous communities need to be studied by historians to determine the ability of written documents to represent the situation.

In summary, we examined the use of NPIs and their effects on the 1918–1920 influenza pandemic in Alaska. These insights provide valuable information to inform pandemic preparedness and management in geographically isolated areas like Alaska.
